# A Novel Role for Triglyceride Metabolism in Foxp3 Expression

**DOI:** 10.3389/fimmu.2019.01860

**Published:** 2019-08-13

**Authors:** Duncan Howie, Annemieke Ten Bokum, Stephen Paul Cobbold, Zhanru Yu, Benedikt M. Kessler, Herman Waldmann

**Affiliations:** ^1^Sir William Dunn School of Pathology, University of Oxford, Oxford, United Kingdom; ^2^Nuffield Department of Medicine, Target Discovery Institute, University of Oxford, Oxford, United Kingdom

**Keywords:** regulatory T (Treg) cell, tolerance, metabolism, differentiation, lipotoxicity, metabolomics

## Abstract

Lipid metabolism plays a key role in many cellular processes. We show here that regulatory T cells have enhanced lipid storage within subcellular lipid droplets (LD). They also express elevated amounts of both isoforms of diacylglycerol acyl transferase (DGAT1 & 2), enzymes required for the terminal step of triacylglycerol synthesis. In regulatory T-cells (Tregs), the conversion of diacylglycerols to triacylglycerols serves two additional purposes other than lipid storage. First, we demonstrate that it protects T cells from the toxic effects of saturated long chain fatty acids. Second, we show that Triglyceride formation is essential for limiting activation of protein kinase C via free diacyl glycerol moieties. Inhibition of DGAT1 resulted in elevated active PKC and nuclear NFKB, as well as impaired Foxp3 induction in response to TGFβ. Thus, Tregs utilize a positive feedback mechanism to promote sustained expression of Foxp3 associated with control of LD formation.

## Introduction

Cells of both the innate and adaptive immune system have been shown to be dependent on different aspects of lipid metabolism for their development and function (1). TGFβ-induced generation of FoxP3+ regulatory T-cells (iTreg) is impaired in the absence of exogenous long chain free fatty acids and enhanced in their presence (2), partly due to Treg preference for FA as an OXPHOS substrate, and partly due to their greater resistance to lipotoxicity (3). In contrast, Th17 cells require endogenous fatty acid synthesis for their development as inhibition of acetyl-CoA-carboxylase, a key enzyme in FA synthesis, favors development of Treg over Th17 (4). Innate immune cells also are dependent on aspects of lipid metabolism for their differentiation. Generation of free fatty acids derived from lipolysis of lipid droplets (LDs) via autophagy in neutrophils is essential for their development from myeloblasts (5). Pro-inflammatory macrophages (M1) and pro-resolution M2 macrophages have opposing requirements for fatty acid synthesis and catabolism. Factors which promote M1 macrophages also induce FA synthesis (6, 7) whereas anti-inflammatory signals which favor M2 macrophages drive fatty acid oxidation (8, 9).

We recently demonstrated that Foxp3+ T cells have a much greater uptake of long chain fatty acids than conventional T cells (Tconv) (3). We proposed that Foxp3+ T cells are protected from potentially damaging acylated free fatty acids as a result of their increased expression of fatty acid catabolic enzymes and diverse components of the mitochondrial electron transport system. There are three main non-mutually exclusive mechanisms a cell can use to avoid the toxic effects of acylated long chain fatty acids; increased β-oxidation in mitochondria, removal via autophagy, and conversion to triglycerides and storage as LDs (10–14).

LDs are dynamic subcellular organelles whose roles in diverse cellular processes are beginning to be appreciated. LDs, in addition to their role in forming a lipid storage depot for the cell, have also been implicated in sequestering transcription factors from the nucleus (15), generating lipid ligands for transcription factors (16), and acting as assembly hubs for pathogenic viruses (17–19). Memory CD8 T cells, which lack LDs, nonetheless use intrinsic lipolysis of ER-resident triglycerides for their maintenance and function (20). LDs comprise a central core of neutral lipids, predominantly triglycerides, steroyl esters, and retinoyl esters bounded by a single layer of amphipathic phospholipids and proteins of the perilipin, adipophilin, and the tail-interacting protein of 47 kDa (PAT) family (21). LD bud from the endoplasmic reticulum wherein DGAT1 and 2 esterify free acylated fatty acids onto diacyl glycerol forming more inert triacyl glycerols which are stored in the LD core. DGAT1 is responsible for esterifying predominantly exogenously-derived fatty acids into TGs, whereas DGAT2 is thought to have a preference in esterifying endogenously synthesized FA (22).

We reasoned that due to their increased FA uptake and enhanced resistance to lipotoxicity it was possible that the formation and interactions of LD with fatty acids in Foxp3+ T cells also differed from that in resting naïve CD4+ T-cells (Tconv). Significant differences in metabolism and storage of LDs between Foxp3+ T cells and Tconv might offer routes to therapeutic targets.

In this study, we demonstrate that LDs are intimately associated with the protection of T cells confronted with lipotoxic fatty acids. Inhibition of triglyceride synthesis, and LD formation, resulted in enhanced cell death from exposure to long chain saturated fatty acids. We show that FoxP3+ regulatory T cells have enhanced lipid droplet numbers and contain relatively increased amounts of di- and tri-glycerides, fatty acids, and phospholipids compared to Tconv. Inhibition of LD development impaired expression of FoxP3 in conditions that generate iTreg. We show that this is likely resulting from the activation of Protein Kinase C by DAG.

## Results

### Foxp3 Expressing T Cells Have an Elevated Lipid Droplet Content

**Figure 1:**
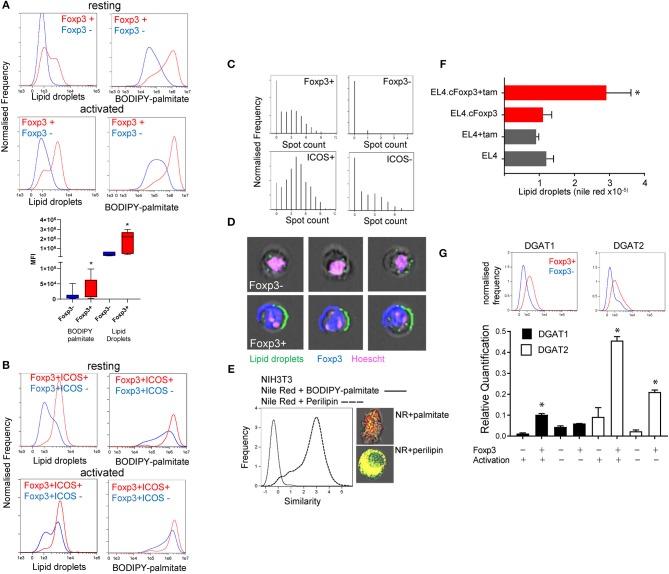
Foxp3 expressing T cells have elevated lipid droplet content. **(A)** Quantitation using Imagestream, of LDs with nile red staining, and palmitate uptake using BODIPY-labeled palmitate on resting and activated Foxp3+ and Foxp3- T cells from the same activation cultures from C57BL/6.Foxp3 hCD2/CD52 knock in mice. The bar chart shows the composite data from 8 separate experiments. ^*^p < 0.05 by ANOVA. **(B)** Measurement using Imagestream, of LDs and fluorescent palmitate uptake in resting, and activated CD4+Foxp3+ICOS+/- cells from the same cultures. **(C)** Top panels; Measurement of LD number in CD4+Foxp3+ and Foxp3- cells using the Imagestream “spot count” feature. Bottom panels; Measurement of LD number in CD4+Foxp3+ICOS+ and ICOS- cells. **(D)** Representative images of Foxp3- and Foxp3+ cells showing bright field, Hoescht, Foxp3, and nile red staining analyzed with Imagestream. **(E)** Measurement of proximity (“Similarity” feature in Imagestream) of nile red staining and perilipin, histogram and bottom image panel, compared to nile red and BODIPY-palmitate, histogram and upper image panel, in NIH3T3 cells. **(F)** Measurement of LD by nile red staining in a tamoxifen-inducible Foxp3 T cell line EL4.cFoxp3 or the parental cell line EL4 in the presence and absence of 4'OH tamoxifen. Error bars represent standard error of the mean, ^*^p < 0.05. Representative of three separate experiments. **(G)** Expression of DGAT1 and DGAT2 protein (top panels) and mRNA transcripts in Foxp3+ T cells and Tconv from B6.KI mice. For mRNA analysis cells were analyzed straight after isolation or after 24 h activation with anti-CD3/28 beads. Error bars represent standard error of the mean, ^*^p < 0.05. Representative of three separate experiments.

We previously showed that Foxp3+ T cells take up significantly more palmitate than Tconv and can use this to fuel OXPHOS (3). This imparts Foxp3+ T cells with a survival advantage in environments with potentially toxic amounts of saturated fatty acids (SFAs). Exogenous fatty acids have been shown to be directed into the triglyceride synthesis pathway for storage in lipid droplets as a protective mechanism to deal with SFAs in non-lymphoid cells (14, 23). We asked whether, in addition to channeling fatty acids for catabolism, Foxp3+ T cells also exhibited elevated LD intensity and numbers. We assessed this in CD4 T cells from Foxp3 reporter mice [B6.RAG^−/−^Foxp3 hCD2/CD52 knock in: henceforth called B6KI (24)]. These mice have a human CD2/CD52 reporter gene knocked in downstream of Foxp3 and cells expressing Foxp3 are identified by surface expression of human CD2 and CD52. We analyzed the LD content and lipid dynamics in mixed populations of Foxp3 positive and negative cells, with minimal intervention, wherever possible in order to avoid potential artifactual metabolic effects due to isolation procedures. Foxp3+ CD4 T cells, either resting or activated by anti-CD3/CD28 coated beads, exhibited higher intensity staining with Nile red, a lipophilic dye specific for LDs, and had greater uptake of fluorescent palmitate (BODIPY FL-C16) (Figure 1A). Effector Treg expressing ICOS (25, 26) had the highest intensity of Nile red staining (Figure 1B). Using Imagestream^TM^ flow cytometry we measured the number of visible Nile Red staining LDs per cell (Figure 1C). Foxp3+ T cells had significantly more LDs than Tconv with a distribution of up to 10 LDs per cell compared to 3 or fewer in Tconv (Figures 1C,D). Similar results were seen when quantifying LDs by staining for perilipin (data not shown). Perilipin staining correlated very closely with Nile red staining, but less so with internalized palmitate which is channeled to mitochondria, discussed below (Figure 1E). We next asked if nuclear Foxp3 expression drives accumulation of LDs using a tamoxifen-conditional Foxp3 expressing cell line EL4.cFoxp3 (3). Tamoxifen treatment of this cell line, but not the parental EL4 cell line, resulted in induction of LDs suggesting that nuclear Foxp3 expression also promotes the expression/generation of factors associated with LD formation (Figure 1F). Expression of Foxp3 does not significantly change in this cell line following tamoxifen exposure (3).

Two rate-limiting enzymes in the production of triglycerides and subsequent LD formation are Diacylglycerol-acyl transferase (DGAT1 and 2) (27). We measured expression of DGAT1 and DGAT2 in flow sorted Foxp3+ T cells and Tconv from B6KI mice using Imagestream^TM^ and quantitative RT-PCR. Foxp3+ T cells expressed higher amounts of both DGAT1 and DGAT2 mRNA transcripts and protein than Tconv (DGAT1mean fluorescence intensity 1531 in Foxp3+ cells vs. 615 in Foxp3- cells, DGAT2 1,306 vs. 493, Figure 1G).

**Figure 2:**
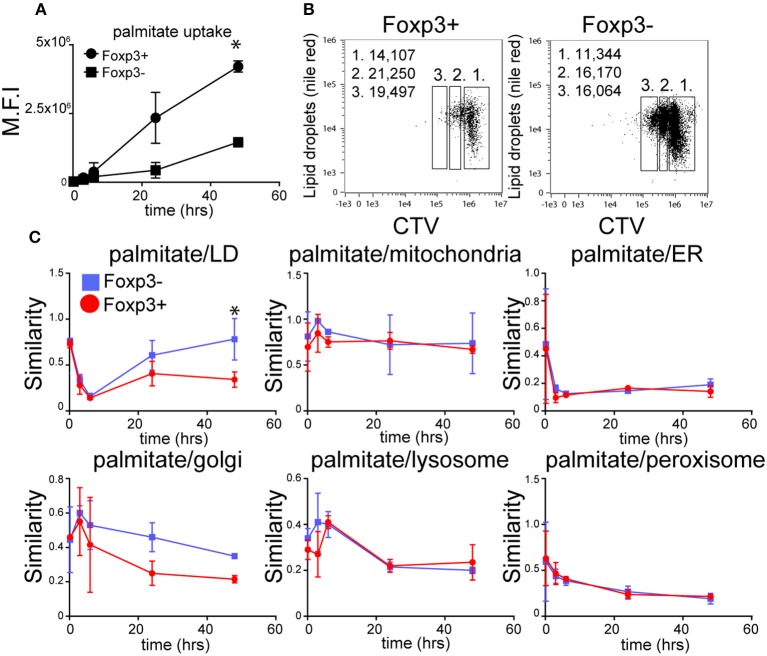
The dynamics of fatty acid uptake and localization differs between Foxp3+ T cells and Tconv. **(A)** Accumulation of BODIPY-labeled palmitate by Foxp3+ T cells and Tconv in mixed CD4 T cell cultures from B6KI mice activated with anti-CD3/CD28 beads for 48 h measured using imaging cytometry. Error bars represent standard error of the mean, ^*^p < 0.05. Representative of three separate experiments. **(B)** Comparison of LD content measured by nile red staining in CD4+ T cells from B6KI mice activated with CD3/CD28 beads for 48 h, stained for Foxp3 and CTV. Left panel; CD4+ T cells gated on Foxp3+ and right panel, Foxp3− stained with nile red and CTV. Gate 1 = undivided, gate 2 = 1st cell division, gate 3 = 2nd cell division. Numbers in the top left of the plots represent mean number of lipid droplets per cell division gate. **(C)** Tracking of BODIPY-labeled palmitate within subcellular compartments of mixed CD4+ T cells from B6KI mice activated for 48 h. BODIPY-palmitate was added at time zero and cells were analyzed by imaging cytometry at 10 min, 3 h, 6 h, 24 h, and 48 h. Cells were stained for Foxp3 and mitotracker to identify mitochondria, nile red for LD, catalase for peroxisomes, CD107 for lysosomes, IP3R1 for endoplasmic reticulum, and Golgin-97 for the Golgi apparatus. Error bars represent standard error of the mean, ^*^p < 0.05. Representative of three separate experiments.

Both Foxp3+ T cells and Tconv in mixed cultures of B6KI CD4+ T cells progressively accumulated palmitate from the cell culture medium during activation (Figure 2A), although Foxp3+ T cells took up ~3-fold more by the end of the culture. LDs also accumulated after activation, with Foxp3+ T cells displaying more LD than Tconv both at the beginning and end of the activation period (Figure 2B). As LD could in principle have been supplied with fatty acids from endogenously synthesized or exogenous sources, we tracked the intracellular fate of palmitate taken up by Foxp3+ T cells and Tconv from the extracellular medium. To this end we took advantage of the ability of imaging cytometry to measure the proximity of adjacent fluorochromes. B6KI CD4+ T cells were bead-activated for 48 h in the presence of BODIPY-palmitate. We calculated a “similarity score” for internalized BODIPY-palmitate's proximity to LD, mitochondria, endoplasmic reticulum, Golgi, peroxisomes, and lysosomes in Foxp3+ and Foxp3- cells at various points following activation (Figure 2C). Similarity score is an in-built feature of the Amnis IDEAS^TM^ software for Imagestream analysis. Surprisingly, although Foxp3+ T cells contain more LD before and after activation, Tconv had a significantly greater co-localization of internalized BODIPY-palmitate with LD than Foxp3+ T cells during the last 24 h of activation. This implies that the palmitate being internalized by Foxp3+ T cells is not being used immediately by these cells to synthesize LDs, but is, presumably, being used in some other way. Palmitate was also detected in proximity to markers for the ER, Golgi, peroxisome, and lysosome in both Foxp3+ T cells and Tconv but no significant differences in similarity scores were seen in these organelles (Figure 2C).

### The Metabolome of Foxp3+ Treg Is Enriched for Lipids

**Figure 3:**
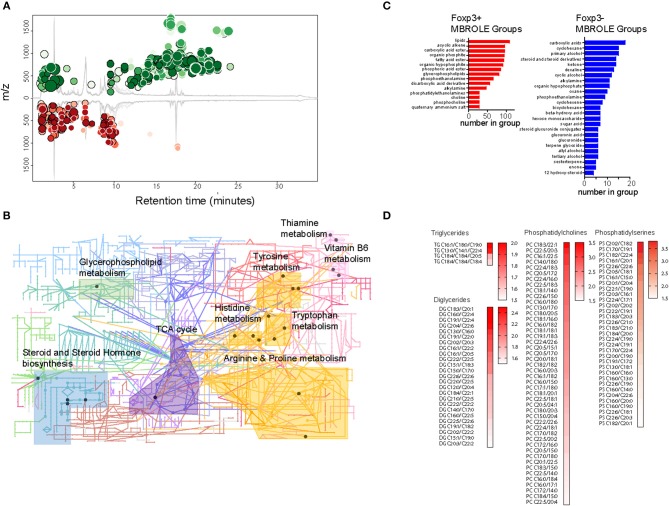
The Treg metabolome is enriched for lipids. **(A)** XC-MS output of 811 significantly increased metabolites detected in Foxp3+ compared with Foxp3- CD4 T cells. Circles represent those metabolites with a fold change of 1.5 or more in Foxp3+ cells and a *p* < 0.01. Radius of the circles correlates to fold change and intensity of color indicates *p* value with deeper color signifying lower *p* value. **(B)** KEGG analysis of representative metabolic pathways enriched in Foxp3+ CD4 T cells. Circles represent nodes of one protein. Dark colored nodes indicate the protein contributes to the significance of the pathway as a whole. Pathway analysis performed using the Mummichog algorithm of Metaboanalyst 4.0 (28). Data from 5 biological replicates of FACS sorted Foxp3+ and Foxp3- T cells. **(C)** Metabolite set enrichment analysis using MBROLE to group significantly overexpressed groups of metabolites in Foxp3+ T cells and groups of metabolites enriched in Foxp3- T cells. Groups shown represent the most significant top and bottom 30% of metabolite sets. **(D)** Heatmaps of triglycerides, diglycerides, and phospholipids present in Foxp3+ cells with a ratio 1.5 or above the expression level in Foxp3- cells. Data represent 5 pooled biological replicates.

To gain insight into the types of lipid enriched in Foxp3+ T cells, we performed a comparative study of the metabolite composition of Foxp3+ T cells and Tconv using quantitative LC-MS QTOF mass spectroscopy. Comparison revealed 15,690 features of which 4,703 could be assigned identities. Firm allocation of molecular identity is difficult for lipids as it is often unknown where carbon double bonds exist on acyl chains, or on which position chemical groups are located on ring structures, for example. XC-MS analysis showed 811 features whose abundance exhibited a >1.5-fold difference between Foxp3+ T cells and Tconv with a *p* < 0.01 (Figure 3A).

We performed metabolite set enrichment analysis using the “Mummichok” algorithm in the Metaboanalyser program to assign raw m/z data to KEGG metabolism pathways (28). The Metaboanalyser program assigns metabolites from MS/MS data to metabolic pathways and maps these to KEGG data to provide a visual indication of metabolic pathways active in cells. 10 KEGG pathways were significantly upregulated in Foxp3+ T cells (Figure 3B). These included “TCA cycle,” “steroid and steroid hormone biosynthesis,” and “Glycerophospholipid metabolism.”

We also used MBROLE analysis (29) to assign individual metabolites into functional or structurally similar metabolic groupings. The top 30% enriched groups in Foxp3+ T cells and Tconv differed greatly in structure. Foxp3+ T cells were enriched for lipids, acyclic alkenes which were predominantly phospholipids, and fatty acids (Figure 3C). Foxp3 negative T cells did not show this accumulation of fatty acids, instead displaying a more diverse range of metabolite classes in the most enriched 30% of MBROLE groups. Foxp3+ T cells displayed enrichment for several membrane components including diacylglycerols, triacylglycerols phosphocholines, and phosphoserines (Figures 3C,D). This is consistent with cytoplasmic accumulations of LDs. Analysis of the degree of saturation of the acyl chains of the constituent lipids in Foxp3+ T cells and Tconv did not reveal a significant bias toward saturated, monounsaturated, or polyunsaturated acyl chains in either subset (Supplementary Figures 1, 2). There were however abundant C16:0 and C18:0 moieties in the acyl chains of DG and phospholipids of both Foxp3 positive and negative T cells (Supplementary Figure 2). C16:0 chains were less abundant in DG and TG of Foxp3 negative cells.

### Foxp3+ T Cells Use LD as a Source of Endogenous Fatty Acids for OXPHOS

**Figure 4:**
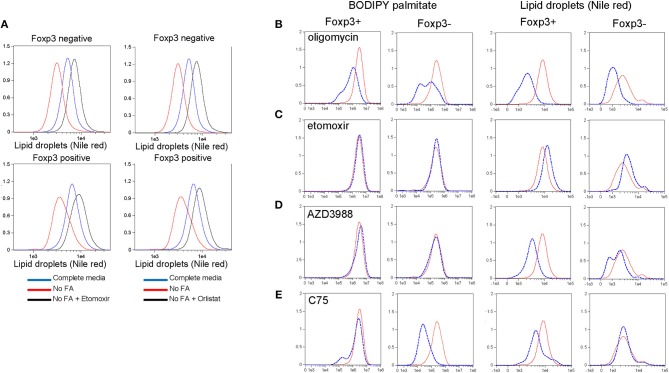
Lipid droplet metabolism in Treg and Tconv. **(A)** Nile red staining in CD4+ T cells from B6KI mice. Cells were cultured for 24 h in complete media (R10), RPMI lacking fatty acids (“no FA”), or media lacking fatty acids and containing Etomoxir or Orlistat. Cells were stained for Foxp3 and nile red. Nile red was quantified on Foxp3 negative and Foxp3 positive cells (top and bottom panels, respectively). Y axes represent normalized frequency. Data representative of two separate experiments. **(B–E)** BODIPY-labeled palmitate uptake and LD accumulation in Foxp3+ and Foxp3- B6KI CD4 T cells treated for 24 h with (dotted blue histograms) or without (solid red histograms) the indicated metabolic drugs. Y axes represent normalized frequency. Data representative of three separate experiments.

LD are dynamic organelles with multiple functions within the cell. They are a storage organelle for lipids (21), a place to sequester potentially damaging saturated fatty acids during autophagy (10) and they act as a platform for many proteins involved in lipid metabolism [reviewed in (30)]. We asked whether Foxp3+ T cells and Tconv use LDs for energy during starvation and whether Foxp3+ T cells, due to their increased number of LDs, had different preferences for endogenous or extracellular fatty acids. B6.KI CD4 T cells were incubated for 24hr in either complete RPMI medium or RPMI lacking fatty acids, and LDs were measured. Both Foxp3+ T cells and Tconv cultured in the absence of fatty acids reduced their LD content, presumably as a result of their catabolism to rectify the deficit (Figure 4A). To test this hypothesis we added Orlistat, an inhibitor of lysosomal acid lipase (LAL) the enzyme responsible for hydrolysis of cholesterol esters and triglycerides delivered to lysosomes via several pathways in the cell [reviewed in (31)]. Orlistat reversed the inhibition of LD development seen under fatty acid starvation conditions in Foxp3+ T cells and Tconv (Figure 4A) supporting the idea that LDs act as a reserve source of fatty acids to T cells in this situation. We next examined the dynamics of LD synthesis and fatty acid uptake and metabolism in Foxp3+ T cells and Tconv. To this end we inhibited key enzymes in these pathways prior to measurement of LDs and BODIPY-FL-C16 uptake. Treatment of B6KI CD4 T cells with oligomycin to inhibit the mitochondrial ATP synthase and hence OXPHOS resulted in a reduction in both LD content and palmitate uptake (Figure 4B). This result supports the notion that mitochondrial respiration signals to the cell to both take up FAs and synthesize LDs. Inhibition of mitochondrial FA uptake with the CPT1A inhibitor etomoxir did not affect the uptake of palmitate by Foxp3+ T cells or Tconv, but significantly increased the amount of LDs in both cell types (Figure 4C). This result suggests that fatty acid catabolism and storage are finely balanced in T cells; inhibition of catabolism results in transfer to, and increased storage in LDs. Inhibition of DGAT1 with the drug AZD3988 resulted in significant inhibition of LDs in both Foxp3+ T cells and Tconv, without any perturbation of palmitate uptake (Figure 4D). A significant difference in the response to inhibition of the fatty acid synthase (FAS) was seen between Foxp3+ T cells and Tconv (Figure 4E). Treg, upon inhibition of FAS using the drug C75, have 40% lower reserves of LDs, whereas Tconv do not reduce LD number. Tconv significantly reduce palmitate uptake on C75 treatment (87% decrease) whilst Foxp3+ T cells display a 43% reduction. These data suggest that Foxp3+ T cells mobilize fatty acids under these conditions from LDs whereas Tconv do not, presumably fueling OXPHOS from alternative carbon sources.

### Intracellular LD Formation Controls T Cell Responses to Lipotoxicity

**Figure 5:**
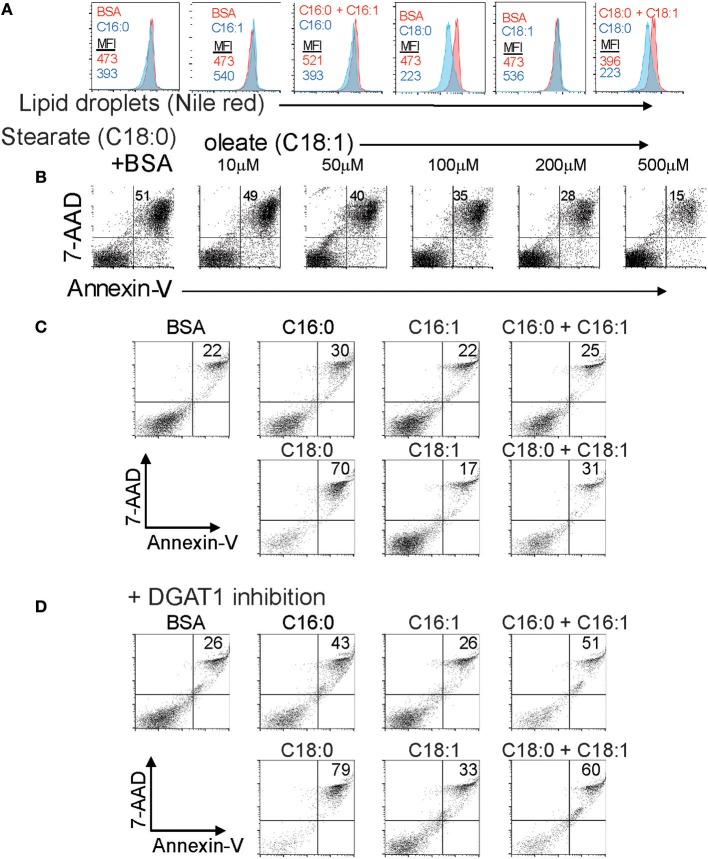
The extracellular ratio of MUFA to SFA and intracellular lipid droplet formation controls T cell responses to lipotoxicity. **(A)** LD content in CD4 T cells exposed to combinations of BSA-conjugated saturated and mono-unsaturated 16 and 18 carbon long chain fatty acids measured by nile red staining. Mean fluorescence intensity values of nile red are displayed for each control (BSA alone), fatty acid, or fatty acid combination. Data representative of three separate experiments. **(B)** Annexin-V-PE and 7-AAD staining of B6KI CD4 T cells cultured for 24 h in media containing 500 μM stearate-BSA alone or stearate-BSA plus increasing concentrations of oleate-BSA. Data representative of three separate experiments. **(C)** Annexin-V-PE and 7-AAD staining of B6KI CD4 T cells cultured for 24 h in media containing 500 μM of the indicated BSA-conjugated fatty acids. Data representative of three separate experiments. **(D)** Annexin-V-PE and 7-AAD staining of B6KI CD4 T cells cultured for 24 h in media containing 500 μM of the indicated BSA-conjugated fatty acids in the presence of 50 μM AZD3988. Data representative of three separate experiments.

LD synthesis is associated with survival of multiple cell types in the presence of elevated intracellular saturated long chain fatty acids derived from the extracellular milieu or from autophagy of triglycerides (10, 14). Our previous work showed that Foxp3+ T cells are resistant to saturated fatty acid-induced lipotoxicity partly through increased β-oxidation of these FAs (3). We asked to what extent formation of LDs in T cells was also associated with survival in the presence of varying ratios of saturated and unsaturated long chain fatty acids. CD4+ T cells were cultured in the presence of saturated LCFAs; palmitate (C16:0) or stearate (C18:0) in the presence and absence of the corresponding monounsaturated FA; palmitoleate (C16:1) or oleate (C18:1). Both saturated forms inhibited the amount of LDs detectable by the intensity of Nile red staining (Figure 5A). The LD content reduction seen after exposure to saturated fatty acids is most likely due to a rise in the ratio of saturated/MUFA which inhibits triglyceride synthesis. In contrast, incubation with the monounsaturated forms (MUFA) alone increased the LD content of the cells. Co-incubation of T cells exposed to saturated fatty acids with monounsaturated fatty acids reversed the decrease in LD content induced by the saturated forms. These data reinforce the idea that monounsaturated long chain fatty acids promote LD synthesis, and are necessary to channel saturated fatty acids into LDs in T cells, a process that has been observed in fibroblasts (14). We next asked whether monounsaturated fatty acid-stimulated LD production was associated with enhanced cell survival in the presence of lipotoxic saturated FAs. CD4 T cells incubated with stearate exhibited 51% Annexin-V/7AAD double positive cells indicative of late apoptosis. Co-incubation with increasing amounts of oleate resulted in protection from apoptosis, with equimolar stearate/oleate resulting in 15% late apoptotic cells (Figure 5B). We tested whether triglyceride synthesis during exposure to equimolar saturated/unsaturated fatty acids was required for protection from apoptosis (Figure 5C). Co-mixtures of the fatty acids protected the cells from palmitate or stearate-induced apoptosis as before (Figure 5C). Treatment of the cells with the DGAT1 inhibitor AZD3988 prevented the protective effect of monounsaturated fatty acids (Figure 5D) showing that monounsaturated fatty acids protect T cells from the damaging effects of saturated fatty acids by facilitating triglyceride synthesis. Given these data we presume that LD production would also be protective of Tregs exposed to lipotoxic saturated FAs, but this has yet to be shown experimentally.

### DGAT1 Facilitates Foxp3 Induction by Limiting PKC Activity

**Figure 6:**
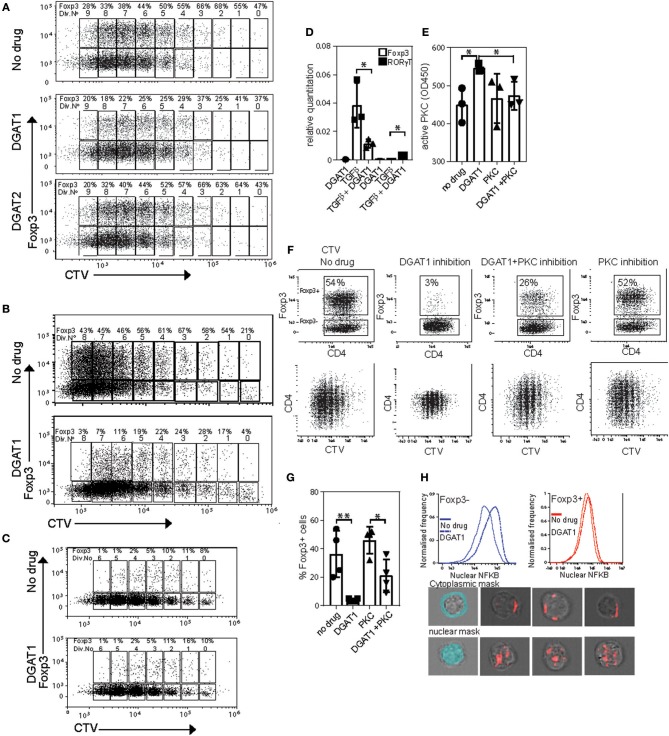
DGAT1 activity diminishes PKC activity and enhances iTreg differentiation. **(A)** Expression of Foxp3 in CD4 T cells from MARKI mice. Cell trace violet labeled cells were activated with CD3/CD28 beads IL2 and TGFβ for 5 days in the presence or absence of 10 μM AZD3988 or the DGAT2 inhibitor JNJDGAT2 at 100 nM. **(B)** Expression of Foxp3 in dendritic cell-activated CD4 T cells from MARKI mice. Cell trace violet labeled CD4 T cells were activated with Dby peptide presented by female C57Bl/6 dendritic cells and TGFβ for 5 days in the presence or absence of 10 μM AZD3988. **(C)** Expression of Foxp3 in CD4 T cells from B6.KI mice. Cell trace violet labeled cells were activated with CD3/CD28 beads and IL2 for 5 days in the presence or absence of 10 μM AZD3988. **(D)** Effect of DGAT1 inhibition on expression of Foxp3 and RORγt mRNA in MARKI CD4 T cells cultured for 5 days in iTreg conditions. Error bars represent standard error of the mean, ^*^p < 0.05. Representative of three separate experiments. **(E)** Quantitation of active Protein kinase C in MARKI CD4 T cells cultured for 5 days under iTreg conditions. Cells were treated with AZD3988 (“DGAT”) with or without the PKC inhibitor Sotrastaurin (“PKC”). Data from three separate experiments. **(F)** Measurement of proliferation and expression of Foxp3 in MARKI CD4 T cells cultured for 5 days under iTreg conditions with the indicated DGAT1 or PKC inhibitors. Results representative of three experiments. **(G)** Enumeration of the effect of DGAT1 and/or PKC inhibition on Foxp3 induction, data from 4 separate experiments. **(H)** Measurement of nuclear NFKB by Imagestream in MARKI CD4 T cells activated for 5 days in iTreg conditions with or without DGAT1 inhibition. Lower panel: Masks for cytoplasm and nucleus (light blue), and representative images of Foxp3 negative cells with cytoplasmic (top) and nuclear (bottom) NFKB staining.

Because we showed that Foxp3 drives upregulation of DGAT enzymes, and that triglycerides are necessary to provide protection from lipotoxic stress, we asked whether LD are required for induction of Foxp3 in response to TGFβ. To this end we used CD4 T cells from Marilyn.RAG^−/−^Foxp3 hCD2/CD52 knock in mice (termed “MARKI” from now on), which have no Foxp3+ T cells, but whose Tconv become Foxp3+ in response to TGFβ (24). CD4+ T cells from MARKI mice were activated with anti-CD3 and anti-CD28 coated beads in the presence of TGFβ and AZD3988 (a selective DGAT1 inhibitor) or JNJDGAT2 (a selective DGAT2 inhibitor) or vehicle control (Figure 6A). DGAT-inhibitory drugs were used at concentrations pre-tested to have no effect on cell division rates (data not shown). Activation of these Foxp3-induction cultures for 5 days resulted in 9 cell divisions in all groups. Almost 50% of the cells started to express Foxp3 before the first cell division. Throughout the cell culture DGAT1 inhibition, but not DGAT2 inhibition, reduced the percentage of cells expressing Foxp3 by between 50 and 95% but did not inhibit cell division. A very similar effect of DGAT1 inhibition was seen when MARKI CD4+ T cells were induced to express Foxp3 by stimulation with dendritic cells and cognate Dby peptide in the presence of TGFβ (Figure 6B). DGAT1 inhibition did not affect expression of Foxp3 in pre-existing Foxp3+ CD4+ T cells from B6.KI mice during activation with anti-CD3 and anti-CD28 coated beads (Figure 6C). The degree of inhibition of Foxp3 induction was variable between experiments and ranged from 90% inhibition to 50% inhibition. The effect was seen in more than 5 Foxp3 induction experiments performed. Analysis of mRNA transcripts from cultures of TGFβ-stimulated MARKI CD4 T cells showed that DGAT1 inhibition resulted in a significant reduction of Foxp3 transcripts and a small but significant increase in RORγt transcripts (Figure 6D). However, we saw no evidence of RORγt or IL-17 protein expression changing under these conditions (data not shown).

DGAT1 activity reduces cellular levels of DAG on LD and ER membranes (32), conditions expected to favor protein kinase C inhibition. We therefore asked whether active PKC levels were raised in cells with inhibited DGAT1. MARKI T cells cultured under the same conditions as (6A) with or without the addition of a pan-PKC inhibitor sotrostaurin, displayed ~25% more active PKC following DGAT1 inhibition (Figure 6E). This increase in active PKC was reversed when cells were co-cultured with sotrostaurin in addition to DGAT1 inhibition. The inhibitory effect on TGFβ-mediated Foxp3 induction by DGAT1 inhibition was associated with PKC activation as Foxp3 induction cultures treated with the DGAT1 inhibitor showed around 95% reduction in Foxp3 expression (Figure 6F). Co-culture with sotrastuarin rescued Foxp3 expression compared to DGAT inhibition alone (Figures 6F,G). Inhibition of PKC by itself had little effect on Foxp3 expression as its activity was not significantly raised to begin with. DGAT1 inhibition lead to a small decrease in CD4 expression, the mechanism for this is unknown. DGAT1 inhibition was associated with increased nuclear NFKB localization in both Foxp3+ and – cells (Figure 6H). Thus, DGAT1 activity, but not DGAT2, is necessary for efficient Foxp3 induction in response to TGFβ. The mechanism is due, at least in part, to limiting PKC activity which can inhibit Foxp3 transcription.

## Discussion

Our data supports a positive feedback model in which a transcription factor (Foxp3) programs a metabolic state (LD loading) which in turn imparts stability to the expression of that transcription factor. In this study we have shown that regulatory T cells harbor larger stores of lipids, in the form of di- and tri-glycerides, and phospholipids, and that some of these are sequestered within LDs. We show that these LDs are important in T cells for fuel storage, protection from lipotoxicity and to limit PKC activity during Foxp3 induction.

Our data show clearly that Foxp3 expression induces up-regulation of the two enzymes required for synthesis of triglycerides; DGAT1 and DGAT2. Inhibition of DGAT1 but not DGAT2 inhibited LD production by T cells (unpublished data). As a consequence of this DGAT1 inhibition had more profound effects on *in vitro* iTreg induction than inhibition of DGAT2. DGAT1 and DGAT2, although sharing the same biochemical function, are non-homologous and have widely different substrate preferences and sub cellular positions (22). DGAT1 is localized preferentially at the ER, whereas DGAT2 is present both on the ER and LDs. DGAT1 has a preference for exogenous, over *de-novo* synthesized, fatty acids and may have a greater activity than DGAT2 under conditions of high substrate concentrations. Such conditions may prevail in Foxp3+ T cells which we show take up much larger amounts of fatty acids than Tconv.

Foxp3+ T cells differ from CD4+ Tconv with respect to response to diminished intracellular fatty acid stores. In our experiments Foxp3+ T cells, when exposed to FAS inhibitors reduced their LD numbers and maintained exogenous fatty acid uptake whilst CD4+ Tconv maintained LD numbers but reduced uptake of exogenous fatty acids. We also demonstrated that Foxp3+ T cells, although taking up significantly more palmitate from the culture medium, had less exogenously-derived palmitate associated with LD. This implies both that Foxp3+ T cells more than Tconv, require an uninterrupted fatty acid supply, and that this can be mobilized from LDs on demand. The source of endogenous lipid for LD synthesis in Treg is currently unknown, but may be derived from lipophagy/autophagy. Autophagy has been shown to be required in Treg for their functional and phenotypical stability (33). Future experiments will be required to elucidate the source of these lipids.

We previously reported that Foxp3+ T cells protect themselves from lipotoxic long chain saturated fatty acids through their robustly up-regulated β-oxidation in mitochondria (3). Protection from lipotoxicity can also be achieved through sequestration of such fatty acids in the side chains of di-and tri-glycerides in LD. We show here that T cells adopt this mechanism and require MUFA and DGAT1 in order to license LD production. The mechanism for MUFA-enabled triglyceride synthesis in other cell types has been reported to involve the MUFA-sensor UBXD8 (34). Addition of monounsaturated fatty acid (MUFA) to cells dose dependently protects the cells from cytotoxic effects of SFA by inhibiting FA synthesis and conversion of FAs to TGs stored in LDs. The protective effect of MUFAs in lipotoxicty occurs *via* a mechanism involving the endoplasmic reticulum MUFA sensor UBXD8. UBXD8 blocks TG synthesis by inhibiting conversion of diacylglycerides to TGs. An excess of MUFAs diminishes this inhibition, allowing production of inert TGs, and increasing LD numbers. It seems likely that the same mechanism operates in T cells although this remains to be formally demonstrated.

Lipid catabolism and synthesis has been implicated in the differentiation of Th17 and Foxp3+ T cells subsets. Th17 have been reported to favor a glycolytic/lipogenic mode of metabolism which depends on acetyl –CoA-carboxylase1 (ACC1) an enzyme essential for fatty acid synthesis in these cells (4). Th17 cells require this pathway for membrane phospholipid production, whereas Foxp3+ T cells use exogenous fatty acids for this purpose. Inhibition of ACC1 preferentially favors Foxp3+ T cells expansion and hinders Th17 differentiation (4). Pathogenic Th17, which are potent IL-17 producers, lack expression of a molecule CD5L resulting in raised cholesterol synthesis and gain of RORγt ligands in these cells (35). Additionally, the side chain FA content of the lipids in pathogenic Th17 cells contain more saturated FAs than those in non-pathogenic Th17 cells. We did not detect any differences in the degree of saturation of constituent fatty acyl chains in the lipids of Foxp3+ T cells vs. CD4+ Tconv apart from a small but significant decrease in the level of saturation of the side chains of phosphatidylserines in Foxp3+ T cells (Supplementary Figures 1, 2). However, Foxp3+ T cells had significant over-expression of metabolites associated with multiple metabolic pathways required for lipid synthesis including glycerophospholipid metabolism, TCA cycle, and steroid and steroid hormone biosynthesis. The acyl chains of phospholipids, TGs and DGs of both Foxp3 positive and negative cells were varied with respect to chain length, even and odd chain length and degree of saturation. Analysis of palmitate and stearate side chains in DGs and TGs revealed them to be equally abundant in phospholipids of Foxp3 positive and negative cells. Palmitate was more abundant in the side chains of TG in Foxp3 positive cells than in Foxp3 negative cells. This may be a reflection of the increased abundance of DGAT1 in these cells.

A recent study from Butcher and colleagues (44) showed that memory CD4+ T cells up-regulate DGAT1 message during inflammation. This study also suggested that DGAT1 pharmacologic inhibition and genetic deficiency enhanced Treg numbers within lymphoid and CNS tissues. Their study showed that retinol-dependent Treg induction was enhanced when DGAT1 was inhibited. Possible reasons for this result, seemingly contradictory to our study, are that when retinol is limiting, DGAT1 converts retinol to retinyl esters, effectively sequestering it away from its Foxp3-inducing role. Another possibility is that changes in Treg and Tconv numbers at particular sites during *in vivo* DGAT inhibition might result from disturbances in normal cell migration rather than any effects on cell differentiation. Thus, the balance between TGFβ/retinol-dependent Foxp3 induction and inhibition of PKC activation in generation of Treg might dictate whether DGAT1 has a positive or negative influence on the magnitude of the Treg output. These different roles for DGAT1 will require a more detailed investigation to fully appreciate the underlying processes.

Why might Foxp3+ T cells require large amounts/numbers of LD even in the presence of sufficient exogenous fatty acids? We show here that Foxp3+ T cells reduce their LD content if starved of the ability to synthesize fatty acids, pointing to their use as a source of fatty acids for fuel and the generation of more complex lipids. However, in the steady state Treg take up more exogenous fatty acids than Tconv, and these do not localize to LD to the same extent as Tconv. This observation argues against the idea that LD in Treg are present purely to fuel OXPHOS. LD have been ascribed a variety of functions distinct from their storage role. Foxp3+ T cells have been reported to perform more autophagy than Tconv (33). It is possible that LDs in Foxp3+ T cells have a role in sequestration of toxic fatty acids released upon autophagy or lipolysis, in a similar fashion reported for adipocytes (13). LD have been described as sites for sequestration of nuclear proteins such as the osmo-protective transcription factor NFAT5 in adipocytes (15), the interaction of which with LD would control the response to environmental stress. LDs are also enriched for oxysterols that serve as RORγt ligands which if sequestered in the core of LDs could prevent RORγt activation and Th17 differentiation. The requirement for DGAT1, but not DGAT2 by Foxp3+ T cells for continued Foxp3 expression supports the idea that DGAT1 is involved in increasing the ratio of TG:DG, limiting PKC activation under high intracellular concentrations of internalized FA. In turn this promotes transcription of Foxp3 and differentiation of Treg. This represents a positive feedback pathway such that Foxp3 promotes production of triglycerides which in turn results in enhanced Foxp3 expression. Different roles for NFKB activity on thymic and peripheral Foxp3 induction have been reported. Stimulation conditions which are inhibitory to Foxp3 induction in the periphery, such as high dose TCR engagement require NFKB activation for the Foxp3 inhibition (36). On the other hand constitutive NFKB activity has been shown to enhance thymic Foxp3 expression (37). We think that, in our present study, the former effect applies, such that NFKB induced by LD inhibition leads to the diminution of Foxp3 transcription (36).

These results highlight previously unknown functions for LD in T cells: protection from lipotoxicity and inhibition of PKC activation. The mechanisms by which LD influence PKC activity and Foxp3 transcription remain to be further dissected, when they may provide novel targets for therapeutic manipulation of the immune system.

## Materials and Methods

### Animals and Cells

Mice were bred and maintained in SPF conditions at the Sir William Dunn School of Pathology. All procedures were conducted in accordance with the Home Office Animals (Scientific Procedures) Act of 1986. Marilyn.RAG^−/−^Foxp3hCD2/CD52knock in (MARKI) and C57BL/6.Foxp3 hCD2/CD52 knock in (B6KI) mice have been described previously (24). “Untouched” splenic CD4+T cells were isolated by magnetic sorting using commercial kits (Miltenyi Biotech). iTreg were generated from MARKI mice by culturing CD4 T cells with anti-CD3/CD28 coated beads (Dynabeads mouse T activator, Life Technologies) along with 10 ng/ml of both TGFβ and IL-2 (Peprotech). Activated cells (Tact) were cultured in the same way with the omission of TGFβ. EL4.cFoxp3 cells were generated by transfecting EL4 cells with a construct encoding GFP-Foxp3-ERT fusion protein, previously described (38, 39). EL4.cFoxp3 cells were treated with 50 ng/ml 4'OH tamoxifen to induce nuclear Foxp3 accumulation.

### Staining Cells for 10 Color Flow Cytometric Imaging

In pursuing metabolic studies on T cell populations, we chose where possible, to adopt a strategy of minimal intervention so as to avoid any artifacts that might be introduced by subset isolation procedures. Flow imaging is permissive for this approach as 12 parameters may be imaged simultaneously, allowing metabolic indicators to be imaged alongside cell surface phenotypic markers. Live cell stains were performed *in situ*, with minimal disturbance of the cells, by adding 0.5 ng/ml Mitotracker DR (Invitrogen M22426) plus 2 μl of live/dead aqua (Life technologies L34957: 1 vial reconstituted in 40 μl of DMSO), together with nile red or BODIPY at 1 μg/ml for LD staining. In some cases, cells were stained with cell trace violet prior to culture (CTV, Thermofisher, C34557). For measurement of palmitate uptake cells were incubated with BODIPY-FLC16 (Thermofisher, D3821) at 1 μg/ml in complete culture medium for various times. These stains were incubated in the dark, in a humidified gassed (5% CO_2_) incubator at 37°C for 30–60 min. Cell surface stains including anti-CD4-APC/Cy7 (Biolegend, 100526), anti-ICOS-PE (eBioscience, 12-9942-82) and anti-human CD2-PE/Cy7 (Biolegend, 309214), anti-CD107b-PE (Biolegend, 1085006) were then performed at 4°C for 30 min along with Hoescht at 1 μg/ml or 7-actinomycin D (7AAD: 10 μg/ml) for nuclear staining. For intracellular stains fix/permeabilization buffer for Foxp3 staining (eBioscience 00-5123-43) was added and incubated at 37°C in the dark for 2 h. 1 ml of 1 × Foxp3 permeabilization buffer (eBioscience 00-8333-56) was then added, the cells were thoroughly re-suspended by vigorous pipetting, harvested, and pelleted for labeling with antibody conjugates. Intracellular stains included; Foxp3-PE/Cy7, RORγt-APC (eBioscience), Perilipin (Abcam, ab172907), DGAT1 (Abcam, ab59034), DGAT2 (Abcam, ab59493),IP3R1-PE (Biolegend, 817701), and Golgin97-PE (CDF4, Thermofisher, A21270). Perilipin, DGAT1, and DGAT2 antibodies were conjugated in house to R-PE using a kit from Abcam (ab102918). Apoptotic cells were detected using an Annexin-V/7AAD kit (Biolegend, 640934). Metabolic drugs used in this study were; AZD2988 a DGAT1 inhibitor (Biotechne, 4837), Etomoxir, a cpt1a inhibitor (Sigma, E1905), Oligomycin, a mitochondrial ATP synthase inhibitor (Sigma, 75351). and C75, a FAS inhibitor (Sigma, C5490). All staining experiments were performed at least three times with biological replicates.

### Imaging Flow Cytometry

Samples were run on a 2 camera, 12 channel ImageStream X MkII (Amnis Corporation) with the 60X Multimag objective and the extended depth of field option providing a resolution of 0.3 μm per pixel and 16 μm depth of field. Fluorescent excitation lasers and powers used were 405 nm (50 mW), 488 nm (100 mW), and 643 nm (100 mW) and the side scatter laser was turned off to allow channel 6 to be used for PE-Cy7. Bright field images were captured on channels 1 and 9 (automatic power setting). Between 10,000 and 30,000 images were acquired per sample using INSPIRE 200 software (Amnis Corporation). Images containing beads were excluded during acquisition as low intensity and high modulation of bright field channels 1 and 9. Images were analyzed using the IDEAS v 6.2 software (Amnis Corporation). Cells were resuspended at 1 × 10^7^ cells per ml for loading onto the Imagestream instrument.

### Analysis of Flow Cell Images

A color compensation matrix was generated for all 10 fluorescence channels using samples stained with single color reagents or antibody conjugate coated compensation beads, run with the INSPIRE compensation settings, and analyzed with the IDEAS compensation wizard. Images were gated for focus (using the Gradient RMS feature) on both bright field channels (1 and 9) followed by selecting for singlet cells (DNA intensity/aspect ratio) and live cells at the time of staining, i.e., live/dead aqua low intensity (channel 8) or low bright field contrast (channel 1).

### Quantitative RT-PCR

RNA was isolated from cells using Trizol (Invitrogen) according to the manufacturer's instructions. cDNA was constructed using Superscript II reverse transcriptase (Life Technologies). qRT-PCR was performed on an ABI7500 fast machine using the following Taqman primer-probe sets from Life Technologies; HPRT1 Mm01545399, CD3γ Mm00438095, RORc Mm03682796, Foxp3 Mm00475164, DGAT1 Mm00515643, and DGAT2 Mm00499536. Quantitation was performed using the method of Pfaffl (40).

### Preparation of Albumin-Bound Free Fatty Acids

Fatty acids (0.2M) in ethanol were diluted 1:25 into phosphate buffered saline pH7.4 containing 20% BSA at 60°C with gentle agitation for 30 min prior to dilution in complete culture medium (RPMI with 10% fetal calf serum, 2 mM glutamine, 1 mM pyruvate plus penicillin, and streptomycin) to the desired working concentration. The molar ratio of fatty acid to BSA was kept at <3 to maintain fatty acid/BSA binding.

### Mass Spectrometry Analysis

LC-MS grade acetonitrile and water were obtained from Merck. 2-Propanol (LC-MS grade) and tert-butyl methyl ether were purchased from Fisher Scientific. Ammonium formate (HPLC grade) and methanol were from Sigma-Aldrich. Analyses were performed using an Agilent 1,290 Infinity Ultra-High Performance Liquid Chromatograph equipped with a quaternary pump delivery system (G4204A), HiP autosampler (G4226A), a column thermostat (G1316C), and fitted with a BEH C18 XP Column (130 Å, 1.7 μm, 2.1 × 150 mm; Waters). The UHPLC system was coupled to a 6,560 Ion mobility quadrupole time-of-flight (QToF) mass spectrometer (Agilent Technologies) equipped with a Jetstream ESI-AJS source.

Metabolites were isolated from T cells according to the method of Yu et al. (41). Briefly, cells were washed twice with ice cold PBS, re-suspended in ice cold methanol and water (1:1 v/v) (400 μl). The suspensions were transferred into a Bead Beater tube (Precellys 24, Bertin Technologies) containing washed glass beads (same volume as cell pellet). Samples were subsequently homogenized in a Bead Beater (Precellys 24, Bertin Technologies) for four cycles (6,500 Hz, 45 s), followed by the addition of 1 ml of tert-butyl ether (MTBE) to extract metabolites. After another cycle (6,500 Hz, 45 s) and centrifugation for 20 min at 13,000 g and 4°C, the organic phase was transferred to a glass vial and dried by vacuum centrifugation (Speed Vac). Samples were re-suspended in 40 μl per 3 million cells of 60% acetonitrile in water (5 mM Ammonium formate). Typically, between 1 and 5 μl of sample material was injected, and the data were acquired in QToF mode using positive electrospray ionization (ESI+). Two reference ions, m/z 121.0508 and 922.0097 were used as internal standards. The Dual AJS ESI settings were as follows: gas temperature: 325°C, the drying gas: 5 L/ min, nebulizer 35 MPa, sheath gas temperature 275°C, sheath gas flow 12 L/ min, Vcap 4.000 V, and nozzle voltage 500 V. The fragmentor of the mass spectrometer ToF was set to 400 V. A total analysis time of 42 min was started by a 5-min isocratic gradient composed with 95% buffer A [60% acetonitrile in water (5 mM ammonium formate)] and 5% buffer B [10% acetonitrile in 2-propanol (5 mM ammonium formate)] with a flow rate of 0.25 ml and was followed by the following steps: 5–7 min 95% −50% A; 7–25 min 50% −5% A; 25–30 min 5% A; 30–30.1 min 5–95% A 30.1–35 min 95% A. The gradient was followed by a 7 min post time to re-equilibrate the column. The raw mass spectrometry data was processed using the MassHunter Workstation software package (Agilent Technologies, version B8.0). MS data was analyzed and quantified using Perseus software (v1.5.4.1) and XCMS Online (42, 43) followed by MBROLE (29) and Metaboanalyst software (28) to group data into biologically functional groupings. The MS data is available from the authors upon request.

### Active Protein Kinase C Assay

Active protein kinase C was measured using a kit from Enzo Life Sciences (Catalog number ADI-EKS-420A) according to the manufacturer's directions. 100 ng of total cell lysate protein was used per well of the assay. Assay plates were read at OD45 using a Biotek microQuant plate reader according to the manufacturer's instructions.

### Statistics

All experiments were repeated at least three times with biological replicates. All statistics were performed as indicated using GraphPad Prism 6 for Windows, version 6.05. Student's *t*-test was used to compare 2 groups; in analysis where multiple groups were compared, ANOVA was performed. Significance was defined as *P* < 0.05.

## Data Availability

The metabolomics data used in this study is accessible through Metabolights; (www.ebi.ac.uk/metabolights/MTBLS1147). The accession number is MTBLS1147. Raw MS files are available upon request from the authors.

## Ethics Statement

All animal procedures were conducted in accordance with the Home Office Animals (Scientific Procedures) Act of 1986 under project license number 30/3060, and the local ethical review panel, the central Committee on Animal Care, and Ethical Review (ACER) at the University of Oxford.

### Author Contributions

DH and HW designed research studies and wrote the manuscript. DH, AT, SC, ZY, and HW conducted experiments. DH, AT, ZY, and BK acquired data. DH, AT, SC, and HW analyzed data.

### Conflict of Interest Statement

The authors declare that the research was conducted in the absence of any commercial or financial relationships that could be construed as a potential conflict of interest.

## Abbreviations

DGAT: diacylglycerol acyl transferase
FA: fatty acid
LCFA: long chain fatty acid
LD: lipid droplet
B6KI: B6. RAG^−/−^Foxp3 hCD2/CD52 knock-in mice
MARKI: Marilyn.RAG^−/−^Foxp3 hCD2/CD52 knock-in mice
MUFA: monounsaturated fatty acid
OXPHOS: oxidative phosphorylation
RORγt: RAR-related orphan receptor g
SFA: saturated fatty acid
Tconv: conventional T cell
Treg: regulatory T cell
iTreg: *in vitro* induced Treg
pTreg: *in vivo* peripherally induced Treg
TG: triglyceride
DAG: diglyceride.
