# Supplementary material for: A Novel Role for Triglyceride Metabolism in Foxp3 Expression
Source: Front Immunol. 2019 Aug 13;10:1860. doi: 10.3389/fimmu.2019.01860 (PMC6701200; doi:10.3389/fimmu.2019.01860)
Supplement: Supplementary file 1 [file Image_1.pdf]

Howie et al Supplementary Figure 1

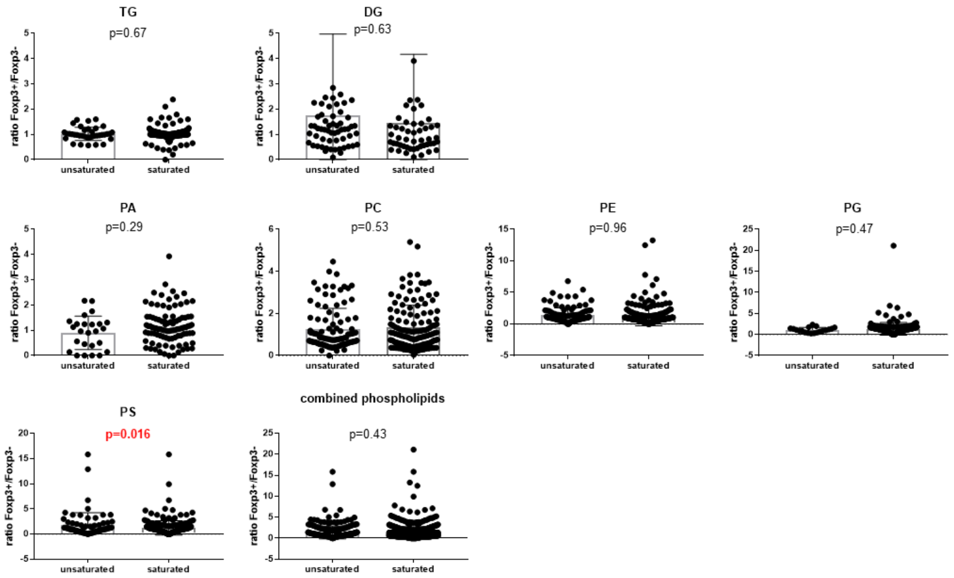

Howie et al Supplementary Figure 1

Ratios of quantity of triglycerides (TG), diglycerides (DG) and phospholipids between Foxp3 positive and negative cells for unsaturated and saturated species. Each dot represents a particular lipid moiety present in both the Foxp3+ and Foxp3- samples. Statistical significance tested by Student's t test. PA=phosphatidic acid, PC=phosphatidylcholine, PE=phosphatidylethanolamine, PG=phosphatidylglycerol, PS=phosphatidylserine. Combined phospholipids panel is the combination of the previous phospholipid data in one comparison.
