# Supplementary material for: A Novel Role for Triglyceride Metabolism in Foxp3 Expression
Source: Front Immunol. 2019 Aug 13;10:1860. doi: 10.3389/fimmu.2019.01860 (PMC6701200; doi:10.3389/fimmu.2019.01860)
Supplement: Supplementary file 2 [file Image_2.pdf]

Howie et al Supplementary Figure 2

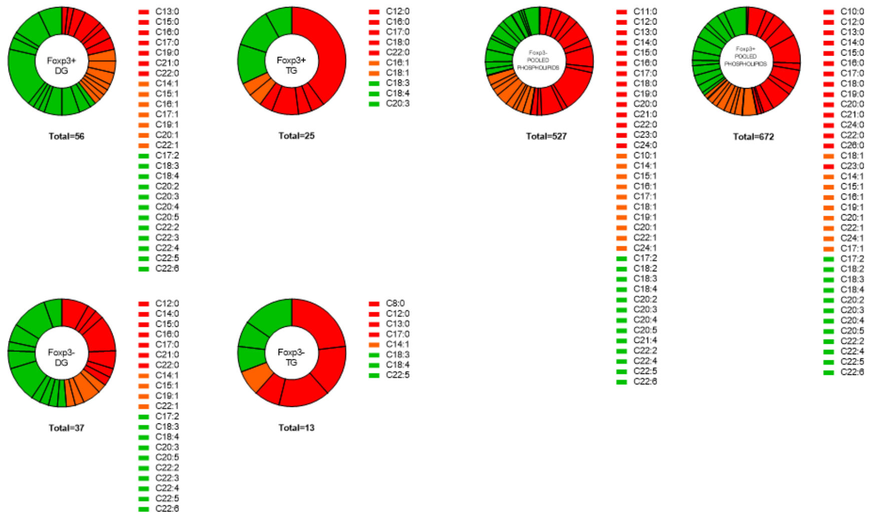

Howie et al Supplementary Figure 2

Distribution of chain lengths and level of saturation of diacylglycerols (DG), triacylglycerols (TG) and pooled phospholipids in Foxp3- and Foxp3+ cells.
